# Navigating AI in Mental Health Care and Psychotherapy: Proposing the GUIDE Framework

**DOI:** 10.2196/101942

**Published:** 2026-09-30

**Authors:** Grant H Brenner, Timothy Gutwald, Jacqueline Ourman, Rachel Wood

**Affiliations:** 1 Brighter NeuroTherapeutics New York, NY United States; 2 Department of Psychiatry and Behavioral Sciences Icahn School of Medicine at Mount Sinai New York, NY United States; 3 Elevare Law Richmond, VA United States; 4 Values Aligned Therapy New York, NY United States; 5 AI Mental Health Collective Colorado Springs, CO United States

**Keywords:** artificial intelligence, AI, mental health, psychotherapy, clinical ethics, informed consent, large language models, digital therapeutics, shared decision-making, clinical framework, harm reduction

## Abstract

AI is already in the mental health consulting room, whether clinicians invite it or not. OpenAI reports more than 800 million regular users of ChatGPT and more than 40 million people turning to the platform daily for health questions, yet the evidence remains limited on the clinical efficacy of large language model–based mental health chatbots. Professional guidance and regulation remain fragmented. Clinicians are therefore practicing in a gap between a patient reality that cannot be ignored and a professional infrastructure not yet built. We argue that neither enthusiastic adoption nor principled abstention is sufficient in the current environment. The clinician who refuses to discuss clinically material patient AI use is not preventing that use; they may lose visibility into a factor materially affecting the therapeutic process. The clinician who adopts AI without structured evaluation exposes patients and practice to avoidable harm. The stance we propose is structured harm reduction within the therapeutic relationship: inviting discussion at intake, integrating material use into case formulation, discussing benefits and limits, establishing crisis boundaries, monitoring throughout treatment, and addressing termination planning. Clinician-initiated use is a separate choice requiring evidence, consent when applicable, privacy safeguards, and accountability. We propose a phased clinical framework spanning preintake to termination anchored by the gather, understand, inform, document, and evaluate (GUIDE) mnemonic as an operational wrapper for daily practice. GUIDE and the phased framework are conceptual, unvalidated proposals. We distinguish ethical recommendations, established legal duties, and predicted standards; scope the legal discussion to the United States; integrate equity and subgroup performance; and offer proportionate implementation. The patients who seek care in an AI-saturated world deserve professional infrastructure capable of holding innovation and patient protection in informed balance.

## The Problem That Clinicians Cannot Avoid

The integration of AI into mental health has already happened. It has happened in the lives of patients ahead of the professional guidance intended to shape it. AI enters care through 4 routes: patient-facing chatbots, clinician-facing scribes and administrative systems, clinical decision support systems, and prescribed or shared digital therapeutics. These routes differ in who initiates and controls the use. OpenAI reports that ChatGPT has more than 800 million regular users worldwide and that more than 40 million people turn to the platform daily for health questions [1]. Clinicians are routinely meeting patients who have consulted an AI before their first appointment or processed difficult material with a chatbot between sessions. Haber et al [2] describe this presence as an “artificial third” in the therapeutic dyad.

The enthusiasm has outpaced the evidence. In a systematic review of 160 mental health chatbot studies, large language model–based chatbots accounted for 16% of the 75 clinical efficacy studies [3]. A systematic review and meta-analysis of 18 randomized controlled trials found short-term improvements in depression and anxiety but no substantial therapeutic effects at the 3-month follow-up [4], leaving open whether short-term benefits translate to durable clinical change. In a preference-based survey of 1612 online participants, respondents with avoidant or anxious-ambivalent attachment and greater symptom burden reported greater acceptance of AI mental health tools [5]. Because the study measured preferences rather than clinical outcomes and may not generalize, the finding is a hypothesis for individualized assessment rather than a settled vulnerability paradox.

Abstention is not neutral. A clinician who declines to discuss clinically material patient AI use does not prevent the patient’s engagement with it; they may lose visibility into an influence shaping the clinical picture. Refusal to discuss AI use does not make it absent—it makes it less available for collaborative examination. The responsibility to address AI-related factors follows from the reality that the technology is already a clinical variable. We propose structured, boundaried harm reduction, not clinician adoption or an established standard of care.

## Why Existing Guidance Falls Short

The gap between position and practice is the defining feature of the current professional landscape. Major organizations have acknowledged the stakes. The American Psychological Association affirms that clinicians retain responsibility and that AI should augment rather than replace clinical judgment [6]; the American Psychiatric Association app evaluation model provides a hierarchical review structure for individual tools [7]. These are valuable contributions. They do not tell the clinician what to ask at intake, how to document a decision about clinically material chatbot use, or how to monitor as AI tools evolve. Many concerns related to AI have analogues in internet health searching, teletherapy, wellness applications, and online peer support; generative AI intensifies them through adaptive dialogue, personalization, opacity, and scale.

At the regulatory level, state legislatures are moving unevenly. A 50-state review found limited clinical and patient input in most proposed legislation, leaving clinicians without consistent guidance and patients without coherent protection [8]. Where requirements or guidance have emerged, they remain jurisdictionally narrow: the Illinois Wellness and Oversight for Psychological Resources Act imposes specified limits and consent requirements [9], New Mexico addresses counselors’ and therapists’ use of AI [10], and Utah provides nonbinding practice guidance [11]. The practitioner crossing state lines or treating patients who do faces a patchwork.

Meanwhile, products move faster than professional guidance. Availability, ownership, functions, evidence, privacy terms, and data practices can change. The tool that a clinician considered in spring may be different or unavailable by fall. What the field requires is guidance tied to the clinician’s decision points, not to the marketplace’s current inventory.

## Five Questions Before Engaging With an AI Tool

Before integrating any AI tool into clinical practice, recommending one, or discussing a tool already in use, 5 questions, summarized in Table 1, warrant consideration. Within the gather, understand, inform, document, and evaluate (GUIDE) mnemonic, these form a tool-level check in the “understand” step. GUIDE is a conceptual framework that has not been empirically validated and is not intended to establish a mandatory standard of care. Only those relevant to the use class should be applied. Patient-selected tools may be discussed without implying endorsement; clinician-selected tools require proportionate due diligence. Review should include cultural and language fit, accessibility, subgroup performance, differential errors, and appeal or escalation mechanisms.

**Table 1:** Five questions to ask before adopting, recommending, or discussing an AI tool.

Question	What to verify
What function and evidence claims are being evaluated?	Identify the use class, intended function, population, and outcome. Does the available evidence match the claimed use? Regulatory authorization or evidence from randomized controlled trials are relevant only where applicable; “AI powered” is not a clinical designation.
Where do the data go, and who controls them?	What is collected, stored, shared, or reused for training? For clinician-selected tools, verify applicable privacy, business associate, and psychotherapy note segregation requirements. For patient-selected consumer tools, discuss known limits without implying that the clinician audited the product.
What is the continuity and exit plan?	Do not attempt to predict company survival. Determine whether data can be exported or deleted; whether alternatives exist; and how care will continue if ownership, terms, functions, evidence, or availability change.
Is the tool appropriate and equitable for this person and use?	Consider clinical, cultural, and linguistic fit; accessibility; digital literacy; and available performance or error data for relevant subgroups. Ask whether differential harms are monitored and whether users can contest or escalate problematic outputs. Treat the cited preference survey as a prompt for individualized inquiry, not outcome evidence [5].
How will benefit, harm, and stopping be recognized?	Agree on observable outcomes; a review interval proportionate to the risk; crisis and escalation boundaries; material change triggers; and stopping, tapering, transition, or re-engagement criteria.

## A Phased Clinical Framework

### Overview

The phased framework presented in this paper anchors clinical responsibility to the structure of the treatment relationship rather than to any particular tool. It spans 5 phases—preintake, intake and evaluation, treatment agreement, ongoing care, and termination—each with concrete questions that the clinician may consider. The framework is product agnostic by design. Tools will change, the decision points will not. GUIDE operates across phases, the 5 questions sit within GUIDE’s “understand” step, and the legal tiers inform GUIDE’s “inform” and “document” steps. Mapping responsibility across the treatment life cycle is the proposal’s distinct contribution.

### Preintake

Clinical responsibility begins before the therapeutic relationship is formally established, but clinicians can govern only their own practice and conditions of access. Public-facing communications—websites, intake paperwork, and initial screening—should identify material clinician-facing AI, state that the clinical relationship is human led, and explain when consent will be sought. Individualized assessment belongs at intake.

The practitioner’s intended use of ambient scribing, decision support, or administrative automation should be described before activation, together with applicable privacy and consent information and a non-AI alternative where feasible. The disclosure should be specific enough for patients to understand the tool’s role without implying that every administrative technology carries the same clinical risk.

### Intake and Evaluation

AI use should be assessed at intake with the same clinical seriousness applied to other factors that may shape care: frequency, purpose, emotional significance, and the role the patient assigns to AI in decision-making. The following is a nonjudgmental prompt: “Many people use chatbots, applications, or AI for information, support, journaling, or decisions. Have any been important to you—helpful, unhelpful, or mixed? Would you like us to discuss them as part of your care?” It should be explained that disclosure is voluntary and intended to support care rather than police lawful behavior. Questions should be asked about conflicting advice and effects only when clinically relevant; access to private conversations should not be required.

AI use should then be integrated into case formulation when clinically relevant, where it may function as either therapeutic aid or clinical contraindication. For some patients, structured applications provide genuine scaffolding for executive function, habit formation, stress management, journaling, or psychoeducation. For others, AI engagement may reinforce social withdrawal or substitute algorithmic agreement for human connection. Formulation should distinguish consumer wellness or resilience use from an intervention claiming to assess, treat, or manage a mental disorder. Either may help or harm depending on function, evidence, patient goals, access, and context.

The central question is whether AI engagement is functioning as a bridge toward therapeutic goals or a detour around them. We use “pseudointimacy” for felt closeness produced by emotionally responsive output without a reciprocal human relationship [12]. Hallucinated content [13], dependence on social chatbots [14], and the absence of professional supervision and therapeutic alliance [15] warrant attention. We use “digitizing loneliness” for simulated connection that reproduces rather than relieves isolation [16] and “pseudoempathy” for empathic-seeming language without human understanding or accountability [17]. Multimedia Appendix 1 supports individualized review; the same tool may help one patient and be contraindicated for another.

### Treatment Agreement

The treatment agreement formalizes the clinical and ethical structure for AI involvement across the course of care. The governing principle is human oversight: AI may inform, supplement, or assist; the clinician retains professional responsibility for clinical decisions and for tools that the clinician selects [18,19]. Responsibilities should be distinguished by use. Clinicians should disclose material uses of scribes, decision support, or automation and obtain consent when required by law or context.

Several components warrant explicit address. The clinician’s own material AI use should be disclosed in specific terms. Patient AI use should be approached through a collaborative invitation—not a reporting mandate—to bring significant AI-derived insights or guidance into the therapeutic relationship when the patient considers this helpful. The informed consent discussion should outline constructive uses, limits, crisis boundaries, privacy, and how either party can revisit the plan rather than functioning as a warning document only. Discussing a patient-selected tool does not make the clinician its guarantor or imply endorsement.

### Ongoing Care

Active treatment requires sustained attention to how clinically material AI involvement evolves and whether it continues to serve clinical goals. AI should be revisited when relevant or at an agreed interval, asking collaboratively about changes in benefit, distress, dependence, conflicting advice, accessibility, or goals. Selected content should be reviewed only with permission. The guiding principle is integration rather than substitution: AI-generated material may be brought into the therapeutic relationship as content for collaborative exploration, not treated as a parallel process running alongside the work. Patient-chosen tools remain the patient’s choice; harm reduction engagement is neither recommendation nor certification.

Two monitoring priorities warrant emphasis. The first is clinical effect, including alliance interference, avoidance, deterioration, dependence, or differential errors. The second is material product change when the clinician selected or recommended the tool. Privacy policies, ownership, features, and availability can shift, but no individual clinician can continuously audit the market. Trusted professional updates, organizational resources, supervision, or periodic review scaled to risk should be used, with reassessment after material changes.

### Termination

As treatment concludes, the focus shifts to equipping the patient to sustain therapeutic gains independently. Termination planning should address treatment and any material AI support. For clinician-selected or shared tools, end-of-use criteria; whether use will stop, continue, or taper; transition to non-AI or human supports; warning signs prompting reassessment or re-engagement; and a pathway back to care should be specified. What data can be exported, retained, or deleted and who must act should be reviewed.

Recommendations should be framed clearly and with vulnerability in mind. Any posttreatment recommendation should match the evidence and risk, distinguish wellness support from treatment, and include crisis limits. The American Psychological Association health advisory supports adjunctive rather than substitutive use [20]. Because the durability of AI-assisted benefit is unresolved [4], what will be monitored, when use should stop, and how the patient can return to formal care should be recorded.

## GUIDE: An Operational Wrapper for Clinical Practice

### Overview

The GUIDE mnemonic operationalizes the phased framework as a rapid clinical reference applicable whether the clinician is evaluating a tool for their own workflow, responding to a patient’s existing AI use, or considering a shared digital therapeutic. GUIDE is not a substitute for clinical judgment; it is a structured prompt for exercising it. Table 2 summarizes the framework.

**Table 2:** Gather, understand, inform, document, and evaluate (GUIDE) framework for structured AI-related decisions in therapy.

Step	Core question	Clinical action
Gather	What is the actual use, who controls it, and what is known?	Use neutral, optional inquiry for patient use and disclose material clinician use. Record class, function, frequency, significance, and available source information. Do not demand private conversation logs.
Understand	What are the evidence, risk, equity, and continuity considerations?	Apply the 5 questions: evidence; data; continuity; cultural, linguistic, and accessibility fit; subgroup performance and differential errors; appeal or escalation; and monitoring. Mark unknowns explicitly.
Inform	What choices, limits, and uncertainties should be discussed?	Discuss benefits, risks, alternatives, crisis limits, data use, and whether the tool is patient chosen or clinician selected. Use shared decision-making. Engagement is not endorsement. The legal tiers identify applicable disclosure categories.
Document	What does the law require, and what record is proportionate?	Use the legal tiers while distinguishing established requirements, emerging practice, and authors’ recommendations. Record disclosure, consent, rationale, and clinically material decisions only as applicable.
Evaluate	Is use serving agreed goals, what has changed, and when should it stop?	Track agreed outcomes and material changes; use risk-proportionate review, escalation, stopping, tapering, transition, and re-engagement criteria.

### Proportionate Implementation

Implementation should be proportionate to control, risk, and available resources. Core practice asks nonjudgmentally about clinically material patient use, discloses material clinician use, documents material decisions, states crisis boundaries, and identifies high-risk triggers. Expanded practice adds vendor and data review, context-appropriate consent, sampling of clinician-tool outputs, and re-evaluation after material changes. System-level governance adds procurement review, subgroup and bias audit, incident response, and continuing monitoring. These are implementation levels, not additional legal or risk tiers; solo clinicians are not expected to reproduce health system functions.

Several complementary frameworks fit within GUIDE: AI Safety Levels–Mental Health (ASL-MH), a non–peer-reviewed framework coauthored by GHB for risk stratification [21]; the Integrated Ethical Approach for Computational Psychiatry for ethical deliberation [22]; and reporting guidance for early-stage AI decision support evaluation [23]. These and other frameworks are summarized in Multimedia Appendix 2. Speculative research directions are discussed in Multimedia Appendix 3, and the 4 tool classes are provisionally mapped to the ASL-MH framework in Multimedia Appendix 4.

## Ethical and Legal Framework

### Overview

The use of AI in clinical settings raises legal and ethical obligations and responsibilities. The legal and ethical landscapes continue to evolve, leaving clinicians with a patchwork of new AI-specific rules and long-standing principles. Considerable uncertainty remains about how patients, courts, and licensing boards will apply them. This section addresses US practice and distinguishes binding law, unresolved allegations, emerging practice, and the authors’ recommendations. Requirements vary by jurisdiction, profession, tool, and workflow; this discussion is not legal advice.

### Core Ethical Principles

#### Nonmaleficence (First Do No Harm)

The introduction of AI into clinical settings can create new risks and amplify existing risks. Protective measures are particularly important for vulnerable populations. Review should consider privacy, biased or differential errors, dependence, crisis failure, the intended population, cultural and language fit, accessibility, subgroup performance, and available appeal or escalation mechanisms. The depth of review should increase with the consequence of error and vulnerability of the population.

These are ethical recommendations, not a claim that every clinician must certify every patient-selected product. Clinical engagement with a patient’s AI use is harm reduction, not endorsement. This is illustrated by a complaint filed in the Superior Court of California, County of San Francisco, on May 12, 2026, and published by the plaintiffs’ counsel, which identifies Leila Turner-Scott and Angus Scott as plaintiffs and OpenAI entities and Samuel Altman as defendants; the public copy does not display an authenticated docket number, and its allegations are not judicial findings [24]. This complaint highlights the alleged harms associated with reliance on chatbot advice by someone alleged to have a substantial history of substance use, demonstrating the need to design AI technologies with vulnerable populations in mind.

#### Beneficence (Do Good)

It is not enough to do no harm; AI technology should lead to improved therapeutic outcomes and benefits. Efficiency alone does not establish clinical value. An AI scribe may make a clinician more efficient, but if it degrades the therapeutic relationship, distracts from the encounter, or produces inaccurate documentation, the clinician must balance these benefits and downsides and ensure that AI enhances rather than replaces the therapeutic relationship. Evaluation should also consider autonomy and equity.

When clinicians introduce material AI use into care, we recommend explaining the tool’s role, data handling, human oversight, material limitations, and alternatives and obtaining consent when the applicable law or professional rules require it. Naturally, this requires clinicians to understand the tools they choose well enough to answer basic patient questions. Patient-selected AI can be approached with the nonjudgmental prompt during intake and evaluation, documenting only clinically material information.

### Standard of Care Considerations

The standard of care for mental health practitioners using AI remains largely undefined and is fact and jurisdiction specific. Given AI technologies’ novelty and ongoing development, courts, licensing boards, and professional organizations will continue to shape how familiar duties apply. Existing law and rules may govern competence, independent clinical judgment, confidentiality, documentation integrity, nondiscrimination, and informed consent, but their application depends on discipline, jurisdiction, and circumstances.

We do not characterize routine inquiry into every patient’s AI use, continuous auditing of every product, or a particular audit interval as settled nationwide duties. We recommend proportionate review, output sampling, re-evaluation after material changes, and escalation for high-risk errors. Predictions about what courts or licensing boards may later require are emerging practice, not current law.

### Legal Considerations

#### Overview

Just as with ethical considerations, the legal considerations regarding AI will continue to evolve as technologies and patient use change. For US practice, we propose a 3-tier framework: professional compliance, statutory and regulatory compliance, and contractual compliance. The tiers organize the review of legal considerations and inform GUIDE’s “inform” and “document” steps; responsibilities still differ for clinician-selected, shared, and patient-selected tools.

#### Tier 1: Professional Compliance

Tier 1 begins with clinicians considering their professional obligations. They should identify applicable licensing board rules and professional standards, retain final judgment, and verify AI-generated material used in care. For an ambient scribe, this includes reviewing and correcting notes before signing, confirming that psychotherapy notes remain segregated from the general record, and testing that workflow before use. Periodic output sampling and renewed evaluation after significant changes are the authors’ recommendations; frequency depends on risk, use, and applicable rules.

Clinicians should also consider patient AI use when it becomes clinically relevant. This may include collaborative inquiry, crisis boundaries, and documentation of material advice or shared decisions. Policies should distinguish binding requirements from organizational choices and identify responsibility for incident escalation, termination of clinician-selected use, and data export or deletion. Patient honesty should be invited through trust rather than imposed as a reporting requirement.

#### Tier 2: Statutory and Regulatory Compliance

Tier 2 begins by understanding how state and federal laws affect a particular AI use. Clinicians should assess tool function, data flow, and jurisdiction. Under HIPAA (Health Insurance Portability and Accountability Act), a vendor handling protected health information for a covered entity may be a business associate requiring written assurances under 45 CFR (Code of Federal Regulations) 164.502(e) and 164.504(e). HIPAA permits many uses and disclosures for treatment, payment, and health care operations under 45 CFR 164.506; it is not a blanket patient consent rule for every AI scribe. State laws may add requirements [25].

Clinicians must also monitor applicable AI-specific rules while recognizing that they are jurisdiction limited. Effective November 18, 2025, New Mexico rule 16.27.18.24 requires board-regulated counselors and therapists who use AI in practice to address informed consent, confidentiality, competence, transparency, human oversight, bias, reliability, client opt out, monitoring, and legal compliance [10]. It is not a nationwide rule and does not apply to every clinician. Other states may impose different disclosure, consent, confidentiality, or scope limitations [9,11].

In *Saucedo v Sharp HealthCare* [26], the plaintiff alleged that an AI scribe recorded confidential clinical conversations and transmitted them to an external vendor without appropriate consent, while clinical notes incorrectly documented that consent had been obtained. These allegations illustrate the importance of addressing recording, third-party data handling, and accurate consent documentation when introducing AI scribes. The complaint’s allegations are not findings of liability.

Outside the United States, obligations differ materially. The European Union AI Act follows a phased timetable [27], the United Kingdom and Canada provide jurisdiction-specific medical device guidance for AI-enabled software [28,29], and Australia provides professional obligation guidance for clinicians using AI [30]. The portable questions are who controls the tool, what data and clinical functions are involved, what disclosure or consent applies, and who remains accountable; specific answers require local law.

#### Tier 3: Contractual Compliance

This tier applies to clinicians or health systems looking to incorporate AI into their own practice. Contracts do not displace legal duties or professional judgment. Clinicians should review how patient data are collected, stored, used, shared, retained, exported, and deleted; security safeguards; subprocessors; incident and model change notice; and termination assistance. The contract should reflect confidentiality requirements and include a business associate agreement when HIPAA requires one.

Given uncertainty surrounding liability for AI errors, clinicians and health systems should carefully review representations, warranties, liability limits, indemnification, insurance, and foreseeable risks. These are risk allocation recommendations, not universal requirements. Procurement records should document the review, unresolved risks, decision, and conditions for re-evaluation. Clinicians should also review professional liability coverage for relevant exclusions.

## Counterarguments and Tensions Held in View

### The Access Argument

A consistent case for broader AI adoption is that unmet need dwarfs available professional capacity. Cheap, scalable, around-the-clock tools may address gaps that human care will not close soon. This argument has real force. Our response is not that it is wrong but that it is incomplete. Access to an unvalidated tool is not the same as access to appropriate care. Some low-risk supports may expand reach, whereas autonomous diagnosis, treatment, or crisis functions demand stronger evidence and oversight.

### Feasibility and Proportionality

Ethical aspiration is not a workable responsibility unless clinicians can perform it. No solo practitioner can predict vendor survival, audit every model, or continuously monitor the literature. Our claim is proportional: using feasible safeguards for risks and tools that clinicians control, relying on trustworthy pooled resources, and declining clinician-selected uses that cannot be assessed safely. For patient-selected tools, the task is ordinary clinical assessment and harm reduction, not product certification.

### The Paternalism Concern

A related concern is that clinician gatekeeping of AI use is paternalistic, especially for adults exercising autonomous choice about legal, widely available consumer products. This is a principled concern and deserves a principled answer. The framework does not restrict patient AI use; it invites clinically relevant use into the therapeutic relationship. Patients retain autonomy over whether and how they use AI and what they disclose. The framework does not authorize surveillance or forced access to conversations. Clinician-selected tools differ because the clinician controls the exposure and bears direct responsibility.

### Human Capacities on Both Sides

A final tension applies on both sides of the therapeutic relationship. Clinician reliance may contribute to deskilling, never skilling, or misskilling [31-33], whereas patient use may function as a shortcut past the vulnerability, friction, and discomfort that produce developmental change. AI companions may digitize loneliness rather than address it; pseudoempathy may simulate trust without delivering its substance. These risks are plausible and context dependent, not established consequences of every use. The practical question is which human capacities a tool supports, displaces, or leaves unpracticed.

### The Evidence Cuts Both Ways

The durability problem—short-term chatbot benefits have not consistently persisted [4]—is sometimes cited to argue against AI in mental health care altogether. However, the same finding cuts in the other direction: established treatments also show relapse or recurrence in defined contexts [34,35], and short-term symptom relief is not clinically trivial. Similarly, the finding that LLM-based chatbots accounted for 16% of the reviewed clinical efficacy studies [3] underscores the need for routine clinical evaluation of platforms that are de facto being used for clinical or clinical-adjacent purposes, but does not provide a basis for blanket prohibition or broad endorsement.

### Limitations

This Viewpoint is a contribution to an evolving conversation rather than the final word. It synthesizes literature and interdisciplinary reasoning rather than reporting a systematic review. The GUIDE mnemonic and the phased framework are conceptual and unvalidated; no comparative evidence shows that they improve outcomes. The legal analysis is specific to the United States, and the evidence is thin, fast-moving, and sometimes corporate, observational, or dependent on single recent studies. The framework was not coproduced with patients or lived experience partners; future development should include such input and prospective evaluation. The international caveat is illustrative rather than comprehensive.

## What the Profession Needs Next

The framework offered here is a contribution to an evolving conversation. It is not the final word, and the field’s most consequential needs sit beyond what any single framework can provide. Professional organizations should provide maintained, jurisdiction-aware resources: model consent language, competency benchmarks, procurement questions, evidence summaries, equity and incident reporting guidance, and clear divisions of responsibility. Researchers should validate the framework and prioritize long-term outcomes, developmental effects, and patient-led evaluation.

For individual clinicians, the most proximal priority is working clinical literacy—sufficient to evaluate tools within their scope, identify risk, hold informed conversations with patients, and make principled decisions under uncertainty. The appropriate analogy is not AI expertise but functional competence. Whether AI competency becomes a formal continuing education requirement remains a prediction; training should be proportionate to practice and supported by shared professional infrastructure.

## Conclusions

AI has not entered mental health practice from outside; it has emerged inside the lives of patients and in clinician workflows ahead of the professional infrastructure intended to govern it. Those routes create different responsibilities. We argue that clinicians should ask about patient AI use when clinically relevant and invite shared examination without surveillance. When clinicians select AI, they should use proportionate evidence, consent, privacy, oversight, and exit safeguards.

The posture we propose is neither adoption nor avoidance but informed, boundaried engagement. The GUIDE mnemonic and the phased framework are unvalidated proposals, not statements of settled law or mandatory duty. Protecting patients does not mean prohibiting their AI engagement; it means bringing clinically material use into the therapeutic relationship when the patient is willing, where it can be evaluated and contextualized. The patients who seek mental health care in an AI-saturated world deserve practitioners—and professional infrastructure—capable of holding innovation and patient protection in careful, evidence-informed balance.

## Appendix

Multimedia Appendix 1 Clinical boundaries and use guidelines for patient AI use.

Multimedia Appendix 2 Complementary frameworks for AI integration into mental health practice.

Multimedia Appendix 3 Emerging and near-future technologies.

Multimedia Appendix 4 AI tool categories mapped to the AI Safety Levels–Mental Health risk gradient.

## Abbreviations

ASL-MH: AI Safety Levels–Mental Health
CFR: Code of Federal Regulations
GUIDE: gather, understand, inform, document, and evaluate
HIPAA: Health Insurance Portability and Accountability Act

## Data Availability

This Viewpoint reports no original data. All references cited are publicly available through their respective publishers, repositories, or government websites.

## References

[1] (2026). AI as a healthcare ally: how Americans are navigating the system with ChatGPT. OpenAI.

[2] Haber Y, Levkovich I, Hadar-Shoval D, Elyoseph Z (2024). The artificial third: a broad view of the effects of introducing generative artificial intelligence on psychotherapy. JMIR Ment Health.

[3] Hua Y, Siddals S, Ma Z, Galatzer-Levy I, Xia W, Hau C, Na H, Flathers M, Linardon J, Ayubcha C, Torous J (2025). Charting the evolution of artificial intelligence mental health chatbots from rule-based systems to large language models: a systematic review. World Psychiatry.

[4] Zhong W, Luo J, Zhang H (2024). The therapeutic effectiveness of artificial intelligence-based chatbots in alleviation of depressive and anxiety symptoms in short-course treatments: a systematic review and meta-analysis. J Affect Disord.

[5] Békés V, Aafjes-van Doorn K (2026). The most vulnerable are prone to use AI therapists: the role of attachment, epistemic trust, and mental health symptoms in acceptance of digital mental health interventions. Psychother Res.

[6] (2025). Ethical guidance for AI in the professional practice of health service psychology. American Psychological Association.

[7] The app evaluation model. American Psychiatric Association.

[8] Shumate JN, Rozenblit E, Flathers M, Larrauri CA, Hau C, Xia W, Torous EN, Torous J (2025). Governing AI in mental health: 50-state legislative review. JMIR Ment Health.

[9] Wellness and Oversight for Psychological Resources Act, Public Act 104-0054, 104th General Assembly (Ill 2025).

[10] New Mexico Register / Volume XXXVI, Issue 22 / November 18, 2025. New Mexico Register.

[11] (2025). Best practices for the use of artificial intelligence by mental health therapists. Utah Department of Commerce.

[12] Babu J, Joseph D, Kumar RM, Alexander E, Sasi R, Joseph J (2025). Emotional AI and the rise of pseudo-intimacy: are we trading authenticity for algorithmic affection?. Front Psychol.

[13] Hatem R, Simmons B, Thornton JE (2023). A call to address AI "hallucinations" and how healthcare professionals can mitigate their risks. Cureus.

[14] Laestadius L, Bishop A, Gonzalez M, Illenčík D, Campos-Castillo C (2022). Too human and not human enough: a grounded theory analysis of mental health harms from emotional dependence on the social chatbot Replika. New Media Soc.

[15] Coghlan S, Leins K, Sheldrick S, Cheong M, Gooding P, D'Alfonso S (2023). To chat or bot to chat: ethical issues with using chatbots in mental health. Digit Health.

[16] Jacobs KA (2024). Digital loneliness-changes of social recognition through AI companions. Front Digit Health.

[17] Yirmiya K, Fonagy P (2025). Mentalizing without a mind: psychotherapeutic potential of generative AI. J Med Internet Res.

[18] Chandler C, Foltz PW, Elvevåg B (2022). Improving the applicability of AI for psychiatric applications through human-in-the-loop methodologies. Schizophr Bull.

[19] Ruan QN, Hu SQ, ShangGuan ZH, Zhou SM (2026). The augmented clinician as a framework for human-AI collaboration in mental healthcare. Front Psychiatry.

[20] Use of generative AI chatbots and wellness applications for mental health: an APA health advisory. American Psychological Association.

[21] Brenner GH, Appel JM (2025). Toward a framework for AI safety in mental health: AI Safety Levels-Mental Health (ASL-MH). Neuromodec J.

[22] Putica A, Khanna R, Bosl W, Saraf S, Edgcomb J (2025). Ethical decision-making for AI in mental health: the Integrated Ethical Approach for Computational Psychiatry (IEACP) framework. Psychol Med.

[23] Vasey B, Nagendran M, Campbell B, Clifton DA, Collins GS, Denaxas S, Denniston AK, Faes L, Geerts B, Ibrahim M, Liu X, Mateen BA, Mathur P, McCradden MD, Morgan L, Ordish J, Rogers C, Saria S, Ting DS, Watkinson P, Weber W, Wheatstone P, McCulloch P (2022). Reporting guideline for the early-stage clinical evaluation of decision support systems driven by artificial intelligence: DECIDE-AI. Nat Med.

[24] (2026). Turner-Scott v. OpenAI Found., No. CGC-26-636801 (Cal. Super. Ct. San Francisco Cnty. filed May 12, 2026). Tech Justice Law.

[25] eCFR:: 45 CFR Part 164 -- security and privacy. Code of Federal Regulations.

[26] (2025). Jose A. Saucedo v. Sharp HealthCare, et al., No. 25CU063632C (Cal. Super. Ct., San Diego Cnty. filed Nov. 26, 2025). DocumentCloud.

[27] (2024). Regulation (EU) 2024/1689 of the European Parliament and of the Council of 13 June 2024 laying down harmonised rules on artificial intelligence and amending Regulations (EC) No 300/2008, (EU) No 167/2013, (EU) No 168/2013, (EU) 2018/858, (EU) 2018/1139 and (EU) 2019/2144 and Directives 2014/90/EU, (EU) 2016/797 and (EU) 2020/1828 (Artificial Intelligence Act). European Union.

[28] (2025). Software and artificial intelligence (AI) as a medical device. United Kingdom Government.

[29] (2026). Pre-market guidance for machine learning-enabled medical devices. Government of Canada.

[30] (2024). Meeting your professional obligations when using artificial intelligence in healthcare. Australian Health Practitioner Regulation Agency and National Boards.

[31] Monteith S, Glenn T, Geddes JR, Whybrow PC, Achtyes ED, Bauer R, Bauer M (2026). Artificial intelligence and deskilling in medicine. Br J Psychiatry (Forthcoming).

[32] Morgan DJ, Rodman A, Goodman KE (2025). How physicians can prepare for generative AI. JAMA Intern Med.

[33] Abdulnour RE, Gin B, Boscardin CK (2025). Educational strategies for clinical supervision of artificial intelligence use. N Engl J Med.

[34] Lewis G, Marston L, Duffy L, Freemantle N, Gilbody S, Hunter R, Kendrick T, Kessler D, Mangin D, King M, Lanham P, Moore M, Nazareth I, Wiles N, Bacon F, Bird M, Brabyn S, Burns A, Clarke CS, Hunt A, Pervin J, Lewis G (2021). Maintenance or discontinuation of antidepressants in primary care. N Engl J Med.

[35] Vittengl JR, Clark LA, Dunn TW, Jarrett RB (2007). Reducing relapse and recurrence in unipolar depression: a comparative meta-analysis of cognitive-behavioral therapy's effects. J Consult Clin Psychol.

