# Supplementary material for: Navigating AI in Mental Health Care and Psychotherapy: Proposing the GUIDE Framework
Source: JMIR AI. 2026 Sep 30;5:e101942. doi: 10.2196/101942 (PMC13669700; doi:10.2196/101942)
Supplement: Supplementary file 1 [file ai_v5i1e101942_app1.pdf]

Clinical Boundaries and Usage Guidelines for Patient AI Use

This table distinguishes potentially supportive and concerning uses. Consumer wellness or resilience activities—such as general journaling, scheduling, or skills practice—should not be represented as treatment unless the product makes and substantiates a therapeutic claim. Either category can become clinically relevant depending on use, effects, and patient goals. Use the table as a collaborative discussion aid, not a diagnostic instrument.

**Category**	**Potentially supportive use**	**Potentially concerning use**
Wellness and resilience	Using a consumer tool for general stress management, sleep routines, values reflection, or habit support, with realistic expectations and optional discussion in care.	Treating an unvalidated wellness tool as diagnosis or therapy; delaying needed care; or continuing despite worsening distress, compulsive use, or inaccessible or culturally mismatched output.
Psychoeducation	Using AI to define a clinical term or explain a specific coping skill.	Asking AI to diagnose symptoms or provide a second opinion on a clinician's formal diagnosis.
Skill practice	Role-playing a difficult conversation or practicing 'I' statements with a chatbot.	Using AI to avoid real-world social interaction or to replace human peer support.
Self-observation	Using an AI journal to track mood trends or identify triggers for later discussion.	Asking AI to interpret the underlying meaning of dreams or trauma memories.
Distress or crisis	Using AI only to locate and access human resources during crisis.	Relying on a large language model for suicide intervention or counseling during acute crisis.
Homework and administration	Asking AI for a healthy meal plan or help organizing a daily schedule.	Asking AI to complete therapy homework, bypassing the internal work.

Table S1. Clinical boundaries and usage guidelines for patient AI use.
