# Supplementary material for: Navigating AI in Mental Health Care and Psychotherapy: Proposing the GUIDE Framework
Source: JMIR AI. 2026 Sep 30;5:e101942. doi: 10.2196/101942 (PMC13669700; doi:10.2196/101942)
Supplement: Supplementary file 2 [file ai_v5i1e101942_app2.pdf]

Complementary Frameworks for AI Integration in Mental Health Practice

These frameworks address distinct levels of decision-making and may complement, rather than sit inside, GUIDE. GUIDE organizes action across the treatment lifecycle; the Five Questions support its Understand step; ASL-MH stratifies risk; IEACP supports ethical deliberation; DEFT-AI structures supervision; DECIDE-AI assesses reporting; the Three-Tier Evaluation assesses evidence maturity; the App Evaluation Model screens applications; and Augmented Clinician states a human-oversight philosophy.

Framework	Function	Clinical use
ASL-MH [21]	Risk stratification (meta-framework)	Provisionally locates AI uses on a six-level risk gradient and proposes safeguards, consent, and oversight intensity.
IEACP [22]	Ethical decision-making	Five procedural stages—Identification, Analysis, Decision-making, Implementation, Review—support structured ethical deliberation.
DEFT-AI [34]	Supervision	Discuss, Evaluate, Feedback, Teach: structured educational moments after an AI interaction to support critical engagement with outputs.
DECIDE-AI [23]	Reporting standards	Reporting items for early-stage clinical evaluation of AI decision-support systems; supports appraisal of published evidence.
Three-Tier Evaluation [3]	Evidence maturity	Bench, feasibility, and efficacy testing distinguish technical novelty from clinical validation.
American Psychiatric Association App Evaluation Model [7]	Application screening	Hierarchical review of background, privacy and security, clinical foundation, usability, and data integration.
Augmented Clinician [19]	Positioning and philosophy	Positions AI as a transparent aid to clinician capacity rather than an autonomous replacement.

Table S1. Complementary frameworks for AI integration in mental health practice.

Note. ASL-MH = AI Safety Levels—Mental Health; IEACP = Integrated Ethical Approach for Computational Psychiatry; DEFT-AI = Discuss, Evaluate, Feedback, Teach for AI; DECIDE-AI = reporting guideline for early-stage clinical evaluation of AI decision support. ASL-MH is a non-peer-reviewed framework coauthored by GHB and has not been empirically validated.

References

3. Hua Y, Siddals S, Ma Z, et al. Charting the evolution of artificial intelligence mental health chatbots from rule-based systems to large language models: A systematic review. World Psychiatry. 2025;24(3):383-394. doi:10.1002/wps.21352. PMID: 40948070.

7. American Psychiatric Association. The App Evaluation Model. n.d. Accessed August 8, 2026. https://www.psychiatry.org/psychiatrists/practice/mental-health-apps/the-app-evaluation-model

19. Ruan QN, Hu SQ, ShangGuan ZH, Zhou SM. The augmented clinician as a framework for human-AI collaboration in mental healthcare. Front Psychiatry. 2026;17:1729175. doi:10.3389/fpsyt.2026.1729175. PMID: 41847409.

21. Brenner GH, Appel JM. Toward a framework for AI safety in mental health: AI Safety Levels—Mental Health (ASL-MH). Neuromodec Journal. 2025. Accessed August 9, 2026. https://neuromodec.org/2025/10/toward-a-framework-for-ai-safety-in-mental-health-ai-safety-levels-mental-health-asl-mh/

22. Putica A, Khanna R, Bosl W, Saraf S, Edgcomb J. Ethical decision-making for AI in mental health: The Integrated Ethical Approach for Computational Psychiatry (IEACP) framework. Psychol Med. 2025;55:e213. doi:10.1017/S0033291725101311. PMID: 40702980.

23. Vasey B, Nagendran M, Campbell B, et al. Reporting guideline for the early-stage clinical evaluation of decision support systems driven by artificial intelligence: DECIDE-AI. Nat Med. 2022;28(5):924-933. doi:10.1038/s41591-022-01772-9. PMID: 35585198.

34. Abdulnour RE, Gin B, Boscardin CK. Educational strategies for clinical supervision of artificial intelligence use. N Engl J Med. 2025;393(8):786-797. doi:10.1056/NEJMra2503232. PMID: 40834302.
