# Supplementary material for: Navigating AI in Mental Health Care and Psychotherapy: Proposing the GUIDE Framework
Source: JMIR AI. 2026 Sep 30;5:e101942. doi: 10.2196/101942 (PMC13669700; doi:10.2196/101942)
Supplement: Supplementary file 3 [file ai_v5i1e101942_app3.pdf]

Emerging and Near-Future Technologies

Evidence for emerging AI-supported interventions is heterogeneous and should not be read as a progression toward clinical readiness. In an observational UK National Health Service sample of 244 patients, use of a generative AI support tool during group cognitive behavioral therapy was associated with attendance, dropout, and outcomes, but the design does not establish causation.[1] In a 3-arm self-guided cognitive behavioral therapy trial of 1187 participants, empathic-feedback activation was associated with adherence in an exploratory analysis (odds ratio 9.99, 95% CI 5.80-17.21), while the intention-to-treat primary Patient Health Questionnaire-9 outcome did not differ significantly between arms at week 7 or month 3.[2] A separate randomized trial in 995 Israeli university students with psychological distress reported modest symptom and well-being benefits and associations between perceived agent qualities, engagement, and improvement.[3] These findings are tool- and context-specific.

Agentic systems are being explored for administrative workflows and decision support,[4,5] while AI-enabled measurement-based care extends established psychometric practice.[6] Evidence, oversight, and autonomy differ across these applications; none should be described as ready for autonomous clinical care.

Digital twins in mental health are speculative research models: computational representations proposed to update from longitudinal data and simulate treatment-relevant dynamics.[7] Spitzer and colleagues illustrate the working alliance as a coupled dynamical system that might be modeled this way; this is a theoretical framework, not a validated deployment. Questions about consent, identity, bias, data governance, and relational development require research before clinical recommendation.

References

1. Habicht J, Dina L, McFadyen J, et al. Generative AI-enabled therapy support tool for improved clinical outcomes and patient engagement in group therapy: Real-world observational study. J Med Internet Res. 2025;27:e60435. doi:10.2196/60435. PMID: 40063074.

2. So M, Sekizawa Y, Hashimoto S, et al. Effect of AI-based natural language feedback on engagement and clinical outcomes in fully self-guided internet-based cognitive behavioral therapy for depression: 3-arm randomized controlled trial. J Med Internet Res. 2026;28:e76902. doi:10.2196/76902. PMID: 41490574.

3. Shoshani A, Gurfinkel B, Kor A, et al. Efficacy of a conversational AI agent for psychiatric symptoms and digital therapeutic alliance: A randomized clinical trial. JAMA Netw Open. 2026;9(4):e266713. doi:10.1001/jamanetworkopen.2026.6713. PMID: 41979879.

4. Sharma D, Meshkat S, Perivolaris A, et al. Reimagining psychiatric care with agentic AI: Promise, challenges, and a roadmap forward. NPJ Digit Med. 2026;9(1):252. doi:10.1038/s41746-026-02453-4. PMID: 41699048.

5. Angus DC, Khera R, Lieu T, et al. AI, health, and health care today and tomorrow: The JAMA Summit Report on Artificial Intelligence. JAMA. 2025;334(18):1650-1664. doi:10.1001/jama.2025.18490. PMID: 41082366.

6. Lutz W, Schwartz B, Delgadillo J. Measurement-based and data-informed psychological therapy. Annu Rev Clin Psychol. 2022;18:71-98. doi:10.1146/annurev-clinpsy-071720-014821. PMID: 34910567.

7. Spitzer M, Dattner I, Zilcha-Mano S. Digital twins and the future of precision mental health. Front Psychiatry. 2023;14:1082598. doi:10.3389/fpsyt.2023.1082598. PMID: 36993921.
