# Supplementary material for: Navigating AI in Mental Health Care and Psychotherapy: Proposing the GUIDE Framework
Source: JMIR AI. 2026 Sep 30;5:e101942. doi: 10.2196/101942 (PMC13669700; doi:10.2196/101942)
Supplement: Supplementary file 4 [file ai_v5i1e101942_app4.pdf]

AI Tool Categories Mapped to ASL-MH Risk Levels

This mapping is a provisional application of the non-peer-reviewed ASL-MH proposal coauthored by GHB, not a validated risk score. The proposal defines Level 1 as no clinical relevance, Level 2 as informational use only, Level 3 as supportive interaction tools, and Level 4 as clinical adjunct systems [21]. Assign a provisional level to a particular use by considering clinical function, autonomy, effective human review, population vulnerability, evidence, data sensitivity, and consequences of error. The four tool classes below are not themselves levels. Where a class has no natural fit, the cell says so; asterisks mark examples that extend beyond the source's explicit product descriptions.

**Tool class**	**ASL-MH Level 1**<br>**No clinical relevance**	**ASL-MH Level 2**<br>**Informational only**	**ASL-MH Level 3**<br>**Supportive interaction**	**ASL-MH Level 4**<br>**Clinical adjunct**
**Patient-facing chatbots and consumer wellness tools**	General-purpose AI for everyday tasks, with no mental-health function.	Static psychoeducation and resources; no personalized mental-health advice.	Supportive dialogue, mood tracking, or crisis connection, with human oversight; not for high-acuity settings.	Clinician-integrated assessment or intervention adjunct, with validation and licensed oversight.*
**Clinician-facing documentation and administrative tools**	Scheduling or office administration without mental-health functionality or clinical content.	Educational resources or templates, without patient-specific recommendations.	No direct source example: scribing by itself is not a supportive-interaction tool.	Documentation integrated into structured clinical assessment or decision support, with validation and professional review.*
**Clinical decision support**	General-purpose, nonclinical retrieval or writing assistance.	Mental-health literature retrieval without patient-specific recommendations.	No direct fit: supportive conversation is distinct from clinical decision support.	Structured assessment or differential, treatment, and risk support for licensed clinicians, with validation and transparency.
**Prescribed or shared digital therapeutics**	No direct fit when the tool has a mental-health function or therapeutic claim.	Educational content or resources only; no personalized intervention.	Supportive self-help dialogue or mood monitoring with human oversight.	Clinician-supervised intervention used as a clinical adjunct, with validation, escalation, and stopping rules.*

Table S1. Illustrative mapping of four AI tool classes to the proposed ASL-MH Levels 1 through 4.

Note. *The source does not classify every product type shown here. Level 3 calls for human oversight and excludes high-acuity settings; Level 4 calls for licensed-professional use, clinical validation, and algorithmic transparency. For documentation tools, applicable consent, human verification, psychotherapy-note segregation, and risk-based sampling and monitoring of sensitive-data summaries remain relevant. No existing AI system is established here as equivalent to a qualified clinician for autonomous therapy, diagnosis, or crisis assessment. ASL-MH Levels 5 (autonomous agents) and 6 (experimental superalignment) are outside this table [21].

References

21. Brenner GH, Appel JM. Toward a framework for AI safety in mental health: AI Safety Levels-Mental Health (ASL-MH). *Neuromodec Journal*. 2025. Accessed September 28, 2026. DOI pending on source page. https://neuromodec.org/2025/10/toward-a-framework-for-ai-safety-in-mental-health-ai-safety-levels-mental-health-asl-mh/
